# Does Appendectomy Always Relieve Symptoms?

**Published:** 1914-01

**Authors:** 


Does Appendectomy Always Relieve Symptoms? Scudder and Goodall (*Publications of Massachusetts General Hospital*), analyzed the results, a year after operation, in 640 cases of appendectomy, of which 28.8 per cent. were females and 72.2 per cent. males. Post-operatic pain was ascribed to asthesions in 88 cases. They conclude that complete relief occurs in 94 per cent.—which seems very high when we consider the numerous typic cases according to history, which shatter our snap diagnosis by stating that the appendix has already been removed.

